# How to prevent alcohol and illicit drug use among students in affluent areas: a qualitative study on motivation and attitudes towards prevention

**DOI:** 10.1186/s13011-021-00420-8

**Published:** 2021-11-07

**Authors:** Pia Kvillemo, Linda Hiltunen, Youstina Demetry, Anna-Karin Carlander, Tim Hansson, Johanna Gripenberg, Tobias H. Elgán, Kim Einhorn, Charlotte Skoglund

**Affiliations:** 1 STAD, Centre for Psychiatry Research, Department of Clinical Neuroscience, Karolinska Institutet, & Stockholm Health Care Services, Region Stockholm, Norra Stationsgatan 69, SE-113 64 Stockholm, Sweden; 2 Department of Social Studies, Linnaeus university, Växjö, Sweden; 3 Centre for Psychiatry Research, Department of Clinical Neuroscience, Karolinska Institutet & Stockholm Health Care Services, Region Stockholm, Liljeholmstorget 7, 117 63 Stockholm, Sweden; 4 Department of Neuroscience, Uppsala University, Uppsala, Sweden; 5 Psychiatry North West, Region Stockholm, Sollentunavägen 84, SE-191 22, Sollentuna, Sweden

**Keywords:** Alcohol, Drugs, Affluent, Youth, Prevention, Motivation, Attitudes, Intervention

## Abstract

**Background:**

The use of alcohol and illicit drugs during adolescence can lead to serious short- and long-term health related consequences. Despite a global trend of decreased substance use, in particular alcohol, among adolescents, evidence suggests excessive use of substances by young people in socioeconomically affluent areas. To prevent substance use-related harm, we need in-depth knowledge about the reasons for substance use in this group and how they perceive various prevention interventions. The aim of the current study was to explore motives for using or abstaining from using substances among students in affluent areas as well as their attitudes to, and suggestions for, substance use prevention.

**Methods:**

Twenty high school students (age 15–19 years) in a Swedish affluent municipality were recruited through purposive sampling to take part in semi-structured interviews. Qualitative content analysis of transcribed interviews was performed.

**Results:**

The most prominent motive for substance use appears to be a desire to feel a part of the social milieu and to have high social status within the peer group. Motives for abstaining included academic ambitions, activities requiring sobriety and parental influence. Students reported universal information-based prevention to be irrelevant and hesitation to use selective prevention interventions due to fear of being reported to authorities. Suggested universal prevention concerned reliable information from credible sources, stricter substance control measures for those providing substances, parental involvement, and social leisure activities without substance use. Suggested selective prevention included guaranteed confidentiality and non-judging encounters when seeking help.

**Conclusions:**

Future research on substance use prevention targeting students in affluent areas should take into account the social milieu and with advantage pay attention to students’ suggestions on credible prevention information, stricter control measures for substance providers, parental involvement, substance-free leisure, and confidential ways to seek help with a non-judging approach from adults.

**Supplementary Information:**

The online version contains supplementary material available at 10.1186/s13011-021-00420-8.

## Background

Alcohol consumption and illicit drug use are major public health concerns causing great individual suffering as well as substantial societal costs [1, 2]. Early onset of substance use is especially problematic since the developing brain is vulnerable to the effects of alcohol and drugs, increasing the risk of long-term negative effects, such as harmful use, addiction, and mental health problems [3–6]. Short-term consequences of substance use include intoxication [5, 7], accidents [8[, academic failure [9], and interaction with legal authorities [10], which calls for effective substance use prevention in adolescents and young adults. Such prevention interventions may be universal, targeting the general population, e.g., legal measures and school based programs, or selective, targeting certain vulnerable at-risk groups, i.e., subsections of the population [11]. Selective prevention can be carried out within a universal prevention setting, such as health care or school, but also be delivered directly to the group which it aims to target, face-to-face or digitally [12–15].

The motives to use substances are governed by a number of personal, social and environmental factors [16], ranging from personal knowledge, abilities, beliefs and attitudes, to the influence of family, friends and society [17–20]. Cooper and colleagues [21] have previously identified a number of motives for drinking, i.e., 1) enhancement (drinking to maintain or amplify positive affect), 2) coping (drinking to avoid or dull negative affect), 3) social (drinking to improve parties or gatherings), and 4) conformity (drinking due to social pressure or a need to fit in). Similar motives for illicit drug use have been found by e.g. Kettner and colleagues, who highlighted the attainment of euphoria and enhancement of activities as prominent motives for use of psychoactive substances among people using psychedelics in parallel with other substances [22], along with Boys and colleagues [23–25], who reported on changing mood (e.g., to stop worrying about a problem) and social purposes (e.g., to enjoy the company of friends) as motives for using illicit drugs among young people. Additionally, the authors found that the facilitation of activities (e.g., to concentrate, to work/study), physical effects (e.g., to lose weight), and the managing of the effects of other substances (e.g., to ease or improve) motivated young people to use illicit drugs.

Prior research has repeatedly shown that low socioeconomic status is a risk factor for substance use and related problems [26–28]. However, recent research from Canada [29], the United States [30–32], Serbia [33], Switzerland [34], and Sweden [35] suggest that high socioeconomic status too is associated with excessive substance use among young people, although for other reasons [29–34]. Previous research has highlighted two main explanations for excessive substance use among young people in families with high socioeconomic status; *i)* exceptionally high requirements to perform in both school and leisure activities and *ii)* absence of adult contact, emotionally and physically, due to parents in resourceful and affluent areas spending a lot of time on their work and careers [36, 37]. In addition to these explanations, high physical and social availability due to substantial economic resources and a social milieu were substance use is a natural element, may enable extensive substance use among economically privileged young people [30, 38, 39].

In parallel with identification of various groups at risk for extensive substance use, a growing number of young people globally abstain from using substances [1, 40, 41]. By analyzing data derived from a nationally representative sample of American high school students, Levy and colleagues [40] found an increasing percentage of 12th-graders reporting no current (past 30 days) substance use between 1976 and 2014, showing that a growing proportion of high school students are motivated to abstain from substance use. However, while this global decrease in substance use among adolescents is mirrored in Swedish youths, in particular alcohol use, a more detailed investigation shows large discrepancies across different socioeconomic and geographic areas. Affluent areas in Sweden stand out as breaking the trend, showing increasing alcohol and illicit drug use among adolescents [42, 43].

To date, we lack in-depth knowledge of why youths in affluent areas keep using alcohol and illicit drugs excessively. Furthermore, despite implementation of various strategies and interventions over the last decades [14, 44–48], we have yet no clear guidelines on how to effectively prevent substance use in this specific group, although the importance of parents’ role for preventing substance use in privileged adolescents has been highlighted in a recent study [29]. Moreover, despite the fact that attitudes are assumed to guide behavior [49, 50] and consequently the reception and effects (behavior change) of prevention interventions, the knowledge about affluent adolescents’ attitudes toward current substance use prevention interventions remains limited. To our knowledge, the only study exploring adolescents’ attitudes to substance use prevention was carried out among Spanish adolescents who participated in “open-air gatherings of binge drinkers”. The study concerned adolescents irrespective of their economic background and revealed positive attitudes to restrictions for drunk people [19]. Thus, extended knowledge on what motivates young people in affluent areas to excessively use substances, or abstaining from using, as well as their attitudes to prevention is warranted.

In the current study, we aim to explore motives for using, or abstaining from using, substances among students in affluent areas. In addition, we aim to explore their attitudes to and suggestions for substance use prevention. The findings may make a valuable contribution to the research on tailored substance use prevention for groups of adolescents that may not be sufficiently supported by current prevention strategies.

## Methods

A qualitative interview study was performed among high school students in one of Stockholm county’s most affluent municipalities. The research team developed a semi-structured interview guide (supplementary Interview guide) covering issues regarding the individual’s physical and mental health, extent of alcohol and illicit drug use, motives for use or abstinence, relationships with peers and family, alcohol and drug related norms among peers, family and in the society, and attitudes towards strategies to prevent substance use. Examples of interview questions are: *How would you describe your health? Which are the main reasons why young people drink, do you think? How do you get hold of alcohol as a teenager?*

*What do you know about drug use among young people in Municipality X? How would you describe your social relationships with peers in and outside Municipality X?*

The study was approved by the Swedish Ethical Review Authority (dnr. 2019–02646).

### Study setting

Sweden has strict regulations of alcohol and illicit drugs compared to many other countries [45, 46]. Alcohol beverages (> 3.5% alcohol content by volume) can only be bought at the Swedish Alcohol Retailing Monopoly “Systembolaget” by people 20 years of age or older, or at licensed premises (e.g., bars, restaurants, clubs), at the minimum age of 18 years. The use of illicit drugs is criminalized. The study was carried out in a municipality with 45% higher annual median income than the corresponding figure for all of Sweden, along with the highest educational level among all Swedish municipalities, i.e., 58% of the population (25 years and over) having graduated from university and hold professional degrees, as compared with the national average of 26%. Furthermore, only 6.1% of the inhabitants receive public assistance, compared to a national average of 13.4% [51].

### Recruitment

Purposive sampling was used to recruit students from the three high schools located in the selected municipality. Contact was established by the research team with the principals of the high schools that agreed to participate in the study. Information and invitation to participate in the study was published on the schools’ online platforms, visible for parents and students. Students communicated their initial interest in participating to the assistant principal. Upon consent from the students, the assistant principal forwarded mobile phone numbers of eligible students to the research team. Also, students from other schools in the selected municipality were asked by friends to participate and upon contact with the research team were invited to participate. Forty students signed up to take part in the study, of which 20 were finally interviewed, representing four schools (three in the selected municipality and one in a neighbor municipality). Before the interview, informed consent was obtained by informing the students about confidentiality arrangements, their right to withdraw their participation and subsequently asking them about their consent to participate. The consent was recorded and transcribed along with the following interview. Twenty students who had initially signed up were excluded after initial consent due to incorrect phone numbers or if the potential participants were not reachable on the agreed time for participation. The reason for terminating the recruitment after 20 interviewees was based on the fact that little or no new information was considered to occur by including additional participants.

### Participants

The final sample consisted of 20 students. Background information of the participants is presented in Table 1. The group included eleven girls and nine boys between 15 and 19 years of age. Seven participants attended natural sciences/technology/mathematic programs and 13 attended social sciences/humanities programs. Twelve participants lived in the socioeconomically affluent municipality where the schools were located and eight in neighboring municipalities. The sample included three abstainers and 17 informants who were using substances, the latter referring to self-reported present use of alcohol and/or illicit drugs (without further specification). Additionally, 18 of the participants reported that at least one of their parents had a university education.

**Table 1:** Background variables of the informants

		N	%
Age	15	1	5
	16	4	20
	17	5	25
	18	9	45
	19	1	5
Sex	Girl	11	55
	Boy	9	45
Residence	Affluent municipality where the schools were located	12	60
	Surrounding municipalities	8	40
Study program	Social/hum	13	5
	Natural/tech/math	7	35
Substance use behaviour	Informants using substances	17	85
	Informants not using substances	3	15

During April–May 2020, semi-structured telephone interviews with the students were conducted by five of the authors (PK, YD, AKC, TH, CS). The interviewers had continuous contact during the interview process, exchanging their experiences from the interviews and also the content of the interviews. After 20 interviews had been conducted, it was assessed that no or little new information could be obtained by additional interviews and the interview process was terminated. The interviews, on average around 60 min long, were recorded on audio files and transcribed verbatim.

### Analysis

Qualitative content analysis, informed by Hsieh & Shannon [52] and Granheim & Lundman [53], was used to analyze the interview material. To increase reliability of the analytic process, a team based approach was employed [54], utilizing the broad expertise represented in the research team and the direct experience of information collected from the five interviewers.

**Fig. 1:**
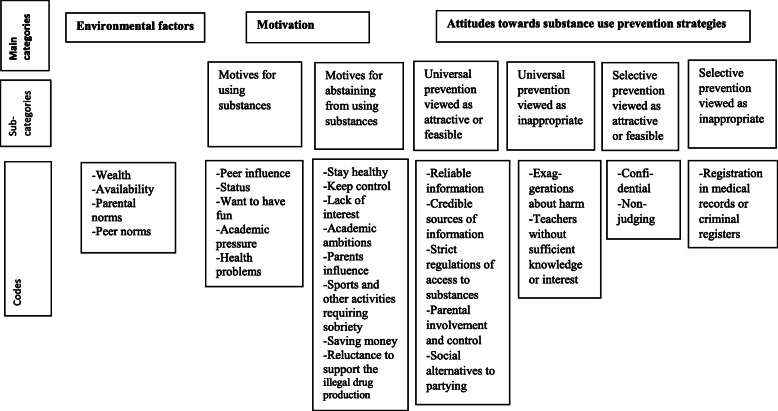
Final coding scheme

The software NVivo 12 was utilized for structuring the interview data. Initially, one of the researchers (PK) read all the interviews repeatedly, searching for meaningful units which could be grouped into preliminary categories and codes, as exemplified in Table 2. During the process, a preliminary coding scheme was developed and presented to the whole research team. After discussion, the coding scheme was slightly revised. Following this procedure, a second coder (CS) applied the updated coding scheme along with definitions (codebook) [54], coding all the interviews independently. Subsequent discussions between PK, YD and CS, resulted in an additionally revised coding scheme. This scheme was utilized by PK and another researcher (LH), who had not been involved in the interviewing or coding, coding all of the interviews independently. The agreement between the coders PK and LH was high and a few disagreements solved through discussion. No change in the codes was necessary and the research team agreed on the coding scheme as outlined in Fig. 1.

**Table 2:** Example of analysis

Meaning unit	Condensed meaning unit	Code	Subcategory	Main category
*But the view is that you cannot have fun without alcohol and therefore, you don’t invite sober people.*	You have to use substances to be invited to parties.	Peer influence	Motives for using substances	Motivation

## Results

The interview material generated three main categories, six subcategories and 27 codes. The results are presented under headings corresponding to the identified subcategories, since they are directly connected to the aim of the study. Content from the main category “External factors” is initially presented to illustrate the context in which the students form their motivation to use or abstain from using substances, as well as their attitudes towards prevention.

### External factors

The external factors found in the interview material concerned wealth, availability of alcohol and other substances, parental norms and peer norms. Informants living in the affluent municipality described an expensive lifestyle with boats, ski trips, summer vacations abroad, and frequent restaurant visits, in contrast to informants from other areas who described a more modest lifestyle. These differences were further accentuated by informants’ descriptions of large villas in the affluent municipality, where students can arrange parties while the parents go to their holiday homes. Some informants further pointed to the fact that people in this municipality easily can afford to buy illicit drugs, increasing the availability.

*The reason why they do it [use illicit drugs] in [the affluent municipality] is because the parents go away, which make it easier to have parties and be able to smoke grass at home, and also because they can afford it*.

(Boy)

Parents’ alcohol norms seemed to vary between families, but most informants described modest drinking at home, with parents consuming alcohol on certain occasions and sometimes when having dinner. However, several informants described that they as minors/children were offered to taste alcohol from the parents’ glasses. Most of the informants meant that their parents trust them not to drink too much when partying.

*They [my parents] have said to me that drinking is not good, but that they understand if I drink, sort of.*

(Boy)

Both parents’ and peers’ norms appear to influence substance use among the students, The impression is that there is an alcohol liberal norm in the local society among adults as well as among adolescents.

*If you want to have a social life in community X, then it is very difficult … you almost cannot have it if you don’t drink at parties.*

(Girl)

### Motives for using substances

Confirming that both alcohol and illicit drugs are frequently used among students in the current municipality, a number of motives for substance use were expressed by the participants. The most prominent motive appeared to be a desire to feel a part of the social milieu and to attain or maintain high social status, with fear of being excluded from attractive social activities and parties if abstaining from substance use. The participants indicated that you are expected to drink alcohol to be included in the local community social life, claiming that this applied to the adult population as well. Alcohol consumption and even intoxication are perceived to be the norm in the students’ social life and several of the participants noted that abstainers risk being considered too boring to be invited to parties.

*The view is that you cannot have fun without alcohol and therefore, you don’t invite sober people.*

(Girl)

There seemed to be a high awareness of one’s own as well as peers’ popularity and social status. Participants evaluated peers as high or low status, fun or boring, claiming that trying to be cool and facilitate contact with others motivates people to use substances. High status students are, according to some participants, frequently invited to parties where alcohol and other substances are easily accessible.

*I would say that our group of friends has more status. […]* *You know quite a few [people] and you are invited to quite a lot of parties. You can often evaluate the group of friends,* *i.e.* *their status, based on which parties they are invited to. […] Some [groups of friends] only drink alcohol and some even take drugs and drink alcohol.*

(Boy)

Some differences in traditions and norms between schools was discerned, with certain schools being especially known for high alcohol consumption and drug use procedures when including new students in the school-community. One of the participants described fairly extensive norm violations, with respect to the law, on these occasions, e.g., strong peer pressure to drink alcohol and use illicit drugs, combined with humiliation of new students, careless driving under the influence of substances with other students in the car, and “punishment” by future exclusion from social events of those who don’t participate at these occasions. On the other hand, already popular, or more senior students, appear to be able to abstain from substance use on occasions without being questioned or risk social exclusion. High self-esteem and a firm approach when occasionally saying no to substances is often respected according to the participants. To avoid peer pressure to use alcohol or illicit drugs, the participants suggested acceptable excuses, such as school duties, bringing your moped or car to the party, having a sports activity or work the day after, or having plans with your parents or extended family during the weekend.

Apart from peer influence, several students expressed hedonistic motives, such as enjoying a nice event or simply to have fun.

*If you want a little extra fun, then you take drugs.*

(Girl)

Apart from social enhancement motives for using substances, some students reported that relaxing from academic pressure or rewarding oneself after an intense period of studying motivates them to use substances. Almost every participant expressed high academic ambitions. One participant who claimed to be very motivated to study expressed drinking due to stress, as illustrated in the extract below:

*You study a lot and you are stressed over school. Then it can be very nice to go out and drink and you can forget everything else for a few hours. […] So it can be a “stress reliever” in that way.*

(Boy)

Yet another participant explained that academic failure had previously made her use substances to comfort herself. Coping with mental health problems, such as depression, was also stated as a reason for substance use. Moreover, some participants reported that they use ADHD (Attention Deficit Hyperactivity Disorder) medication to be able to study more intensively.

### Motives for abstaining from using substances

A number of motives for totally or temporarily abstain from substance use were put forward by the students, such as a wish to be healthy, keep control and avoid embarrassment, influence of parents, academic pressure, sports ambitions or simply lack of interest. Lack of interest in alcohol and drugs was expressed foremost by those attending natural sciences programs and those who totally abstained from substance use.

*I attend the engineering program and I don’t think the interest in alcohol and parties is as present as it might be on social sciences programs.*

(Boy)

Fear of health consequences was predominantly related to abstaining from illicit drugs, but also alcohol. Motives for abstaining from alcohol included perceived risk of being addicted, due to relatives having alcohol problems (heredity), and taking medicine, for example ADHD medicine, since combining alcohol and medication was perceived as risky. Some students had observed friends getting “weird” or “laze” after using illicit drugs, which made them hesitant to use such substances themselves. With regard to parental norms, most parents were by the participants reported to be “normal drinkers” themselves and quite relaxed about their teens’ alcohol consumption. This applied to both the parents of older teens and minors. However, many of the participants reported that their parents would be upset and disappointed if they found out that their child used illicit substances, which motivated some of them to abstain. Reasons for abstaining from substance use included academic strivings, sports performance ambitions, driving, or other activities requiring sobriety, which the students referred to as socially acceptable reason to abstain from substance use. Prioritizing studies over partying was explicitly expressed as the primary motive to abstain by some of the participants.

*We are a group of five or six who come from other municipalities. […] We don’t party and such things and we may be seen as a bit boring. But we are a little more responsible and we are more motivated to study than the others in the class.*

(Girl)

A wish to save money and reluctance to support the illegal drug production were also mentioned as reasons to abstain from substance use, however to a lesser extent.

### Universal prevention viewed as attractive or feasible

With regard to substance information interventions, some students wanted detailed information about different substances’ physical and psychological effects. The participants emphasized the importance of credible sources or persons providing the information, mentioning researchers, young medical students and even parents as credible sources of information. Individuals who had experience of substance use were also suggested.

*You have to tell the facts in a way that makes us want to listen. With the help of various spokespersons who have been involved in it, for example.*

(Girl)

Several students stressed the importance of being able to identify with the person sending the message and suggested influencers as plausible sources. Someone who is difficult to relate to was given as an example of a non-credible, as the following excerpt shows:

*They shouldn’t take a heroin addicts who talk about having found Jesus, because I do not think it would touch the children or touch the young. You have to somehow find … someone that can relate to the young people.*

(Boy)

As for universal prevention, the students also suggested intensified legal measures for companies and people providing young people with alcohol or drugs.

*For example, make it difficult for young people to have access to alcohol [...], allocate more time as a police officer to catch the drug dealers.*

(Girl)

Both alcohol and illicit drugs were reported as easily accessible. Students can obtain alcohol via social media platforms, such as Instagram and Snapchat, where “liquor cars” market themselves and offer home delivery. In addition, older siblings or peers and even some parents were, according to the informants, providing minor students with alcohol. The main way to access illicit drugs is via parties where older students offer drugs to younger peers. Access to prescription drugs was also reported.

Several of the participants agreed that parental involvement is constructive for substance use prevention. Many of them reported having supportive and caring parents involved in their lives, but at the same time referring to friends’ parents as being more absent, resulting in extensive partying in large homes without parental control. Some students reported that parents don’t realize to what extent youths are using substances and that the parents should pay even more attention to what their children do.

*I think [parents should be] keeping track, good track of the kids […]*. *Keeping track of what they are doing and ask them how they feel and things, I think that helps.*

(Boy)

In line with leisure activities as a reason to abstain from substance use, some participants suggested that social activities other than partying could be a way of preventing substance use, as expressed by one participant when asked about plausible ways to prevent substance use.

*Find a sport or friend that you train with […] instead of going to a party,*

(Girl)

Talking about their leisure activities, the participants expressed joy and that these activities made them relax while being social.

*The leisure interests, like working out and hanging out with friends, is relaxing and in contrast to the everyday in some way*.

(Boy)

### Universal prevention viewed as inappropriate

Several of the participants expressed great skepticism towards traditional universal preventive strategies, such as lectures by teachers, social workers or researchers. Some teachers were perceived as ignorant and unengaged, lecturing about substances only by duty.

*The teachers have been a bit like ‘now we’re going to talk about drugs […] and then you have fifteen minutes and they say something like ‘here we are a drug free and smoke and tobacco free school’, and no one obeys.*

(Girl)

Some students also doubted that the information provided from school and society is true, suspecting exaggerated report on harm, and that they prefer information from social media platforms such as Youtube or other online sources.

*It feels like the information we get in school is a bit exaggerated, a bit made up for us […] A bit like this, ‘now we’ll get the young people to stop’.*

(Boy)

### Selective prevention viewed as attractive or feasible

In circumstances where students are worried about their own or peers’ substance use, participants stressed the need for a way to connect with local authority, health care or other support anonymously, without being registered in medical records or being reported to the authorities. Moreover, the participants emphasized the importance of a non-judging approach from professionals when they reach out to students at risk of excessive substance use.

*If you wonder about something or if you are worried about something, then you should be able to turn to adults without being yelled at and know that you are getting positive feedback like ‘I understand you’ and ‘how can we fix this?’ *

(Girl)

### Selective prevention viewed as inappropriate

As indicated above, help-seeking seemed to be counteracted by fear of being recorded in medical records or in the criminal registries. One participant mentioned an incident where a student, caught smoking marijuana, was prosecuted and that this student’s life had been severely affected with cancellation of planned studies abroad and rejection of driving license application. These consequences had, according to the participant, resulted in the student “giving up” and selling illicit alcohol to other students instead of trying to strive for a good future life. Admitting that such an incident can serve as a warning to other students, the fear of consequences is, according to the participant, still an obstacle to seeking help.

*People don’t really know what to do when they see their friends do it [use substances]. You don’t want to tell on them, because they are afraid that if it is written down somewhere, then everything can be ruined.*

(Girl)

Also, parents were by the participants reported as being reluctant to seek help for their children, because of fear of the reporting of their child’s behavior or crime to authorities, with subsequent negative consequences.

*Parents do not dare either because they don’t want it to be about their children. I know some parents who have found drugs in their children’s rooms, but do not want to ruin [future prospects] for them.*

(Girl)

## Discussion

The current study aimed to explore motives for using or abstaining from using substances, including alcohol, among students in affluent areas, as well as their attitudes to and suggestions for substance use prevention.

### Summary of results

The motives for using substances among the students are associated with social aspects as.

well as own pleasure and coping with stressful situations. The most prominent motive appears to be a desire to feel a part of the current social milieu and to attain or maintain high social status within the peer group. Several of the students expressed fear of being excluded from attractive social activities if abstaining from substance use, although some meant that they were not interested in substances and didn’t care if they were perceived as boring, and also had found a small group of friends with whom they socialized. Motives for abstaining, apart from lack of interest, included academic ambitions, activities requiring sobriety, parental influence, and a wish to stay healthy. The students expressed negative attitudes towards current information-based prevention as well as problems with using selective prevention interventions due to fear of being registered or reported to the authorities. Students’ suggestions for feasible universal prevention concerned reliable information from credible sources, stricter substance control measures, extended parental involvement, and social leisure activities without substance use. Suggestions regarding selective prevention were guaranteed confidentiality and non-judging encounters when seeking help due to substance use problems.

### Comparison with previous research

Children of affluence are generally presumed to be at low risk for negative health outcomes. However, the current study, in accordance with other recent studies [29, 55], suggest problems in several domains including alcohol and drug use and stress related problems, even if the cause of these problems cannot be determined based on our interview study. Previous explanations for extensive substance use among affluent young people have been exceptionally high-performance requirements in both school and in leisure activities, and absence of emotional and physical adult contact, resulting from parents in affluent areas spending a lot of time on their jobs and careers [30, 56–58]. These explanations can be viewed in the light of Cooper and colleagues’ [21] as well as Boys and colleagues’ [23–25] previously identified coping motive for substance use. Coping appears among affluent young people as a central motive for substance use, i.e., coping with performance requirements and perhaps with negative affects due to parents’ absence. In the current study, however, social motives, including conformity, i.e., using substances due to social pressure and a need to fit in [21, 23–25] appears to be the most prominent motive, supporting the social learning theory which proposes that behavior can be acquired by observing and imitating others and by rewards connected to the behavior [16, 59]. Interestingly, a small group of participants, especially from natural sciences programs, resisted the general pressure to use substances and found a social context of a few friends with whom they socialized without striving for high social status in the larger social context. The wish to be included in the social life and achieve high social status within the peer group was described as a central motive for substance use among a majority of the students, along with fear of being excluded if abstaining. Previous research show that high socioeconomic status is a protective factor for substance use disorder among adults [60], but among young people it may be the opposite. High status appears to be an important risk factor for the use of substances, at least among those striving for higher status. The students report that they, to achieve high status, must attend parties and at least drink alcohol. After achieving high status, which has resulted in frequent invitations to parties, students then may pose an even higher risk of excessive alcohol and drug use. In line with previous studies, results show that individuals with larger social networks, which has shown to be an indicator for social status among young, also drink more [35, 61]. However, status can also act as a protective factor. Individuals with higher status have, according to the interviewees, slightly more room for maneuver to temporarily say no to substances at a party, without being pressured or ashamed. Nevertheless, several of the interviewees reported that they have to choose between using substances or being excluded from desirable social activities, as abstainers are considered “boring”. The results further show that alcohol and other drugs are popular among affluent youth and the information from the participants indicate that the students perceive substance use to be under control. One possible explanation is that high affluence can contribute to a sense of control over one’s life [62]. Although previous studies show that young people from affluent areas drink more, the risk of developing alcohol problems is still greater among young people who grow up in more disadvantaged areas [57]. Why this is the case is unclear. There is a widespread belief that affluent youngsters have plenty of social and financial resources in the family and thus receive the right help (e.g., psychotherapy) when they have problems [62], which could explain why they do not develop alcohol problems. However, research also shows that parents in affluent areas seek less help than others when their children are troubled [30, 63], partly due to difficulties in accepting and revealing problems within the family [62]. In the current study, the informants expressed doubts about the possibility to be guaranteed confidentiality when seeking help, which may mean that there are concerns among both children and parents about the risk of losing status and a good reputation if seeking help for substance use problems. Consequently, there is a risk that any substance use problems will not be noticed in this group [62].

Previous research indicates that academic pressure may promote substance use [56, 64]. However, in the current study academic pressure, due to high ambitions, was reported both as a reason for using substances and abstaining, the former to cope with stress or relax, the latter to maintain a sharp intellect and receive high grades. Moreover, previous research has demonstrated an association between pressure from extracurricular activities or “over scheduling” and negative outcomes among affluent students (39). In the current study, this did not stand out as a critical vulnerability factor. Instead, students reported extracurricular and leisure activities as relaxing and fun and an accepted reason to abstain from substance use while still attending activities where peers were using substances.

With regard to adult or parental contact, previous research shows that mental health and substance use among adolescents in socioeconomic affluent areas are associated with parents’ lack of reaction to teenage substance use (i.e. liberal, allowing attitudes and minor or no repercussions on discovering use) and parents’ lack of knowledge of their teens’ activities [30]. In our study, the students reported that their parents do not generally react with punishment due to their child’s alcohol consumption. However, the participants thought that parents probably should react more condemningly due to illicit drug use, if revealed. The Swedish criminalization of illicit substance use [46] may influence parents to adopt stricter norms with regard to their children’s illicit substance, because of the consequences for revealed substance use that may occur in the Swedish context. Also, parents in the current study were reported as being reluctant to seek help for their children out of fear of negative consequences that may affect their children. This result is in line with previous research, showing that concern about admitting problems in their children is elevated among affluent parents [30], mentioned above. In the current study, the participants further reported closeness to their parents and that their parents cared about how they spent their time. That said, some parents of wealthy peers were reported as being more absent, resulting in extensive partying in large homes without parental control. Previous research has shown the nature of family relationships and perceptions of closeness to be important protective factors for adolescent mental health [56], and this seems to apply to the students in the current study.

The students’ attitudes to current substance use prevention, aimed to increase students’ knowledge, are to a large extent negative. Information provided in school were reported as exaggerated and uninteresting. Instead, students suggested interventions focusing on credible sources of reliable information, such as from people with personal adverse experiences of substance use and people whom they can identify with. Whether people with own experience of substance use are credible or helpful in a more objective way can be disputed, but the students seem to put their trust in them rather than other persons. This result is partly in line with previous research on school-based programs in general, suggesting that the role of the teacher (the one who deliver the information) is central and that the use of peer leaders can be successful in engaging the students who receive the message [65, 66]. Some informants in the current study meant that the teachers in school were ignorant and unengaged, lecturing about substances only by duty, which of course can be problematic for the sense of credibility among those receiving the information. Previous research has demonstrated that for older adolescents, a social influence approach can increase the effectiveness of alcohol and drug prevention interventions, as can health education, basic skills training and the inclusion of parental support [67]. Again, this research applies to adolescents in general and not to affluent youth specifically.

Interestingly, the students also suggested stricter regulations on substances with intensified legal measures for those providing substances. Positive attitudes to limiting access of alcohol for drunk people have previously been shown in a Spanish study among adolescents participating in an open-air gatherings of binge drinkers [19]. The positive attitude to stricter regulations for those providing substances is interesting in the light of the students’ desire for a non-judging approach when having to seek help for own substance use, as described below. Previous research, however, supports strict policy measures to decrease availability as an effective measure for substance use prevention in the general population [68]. The students further suggested increased parental control and activities and venues which can be attended without using substances, for example sporting/training with friends. Leisure activities without substance use have recently been offered to e.g., adolescents in general in an Icelandic prevention strategy [69], however more research is needed to see if this kind of prevention is attractive also for large groups of affluent students as an alternative to parties and whether it also appears to be effective in reducing substance use in this group. Clearly, some affluent students without ambitions to receive high social status do find socialization without using substances attractive, as shown in the current study. With regard to selective prevention, the students were critical of the current risk of being reported to parents, registered within medical records or reported to the authorities if turning to professionals for support for substance use problems. They claimed that this circumstance serves as a massive counteracting force to seek help at an early stage for oneself or for peers and that the possibility of reaching out anonymously is essential for taking the first step in seeking help. Moreover, the adolescents in this study call for an open and non-judging approach when turning to health care staff, parents or other adults, which is in line with so called Motivational Interviewing, a non-judging approach aimed to enhance motivation to change by exploring and resolving ambivalence about e.g., substance-related behaviors [70], which has shown promising results with regard to reduction of alcohol consumption among young people [71].

### Strengths and limitations

The current study has a number of strengths. Firstly, we were able to recruit both male and female students between 15 and 19 years of age, living inside the affluent community as well as in neighboring municipalities, which provided us with a broad base of the students’ social context. Secondly, we included informants using substances as well as abstainers, increasing the possibility to get a broad view of motives to use or abstain from using substances among affluent youth. Thirdly, the research group has extensive experience in qualitative analysis as well as working with adolescents and young adults with mental health problems, including alcohol and drug consumption or abuse. However, our study must also be viewed in the context of some limitations. Students with more severe health or psychosocial problems may have refrained from participating, biasing the results towards adolescents of more stable psychosocial functioning. Moreover, interview studies are always vulnerable for social desirability bias due to a potential desire to give socially acceptable answers [72]. However, the possibility to terminate participation at any time, along with the circumstance that most of the interviewers are health care professionals, thereby used to handle secrecy in consultation situations, may have decreased the risk of desirability bias in the current study.

## Conclusions

Several of the motives guiding substance use behavior among young people in general also seem to apply to affluent youth. A desire to feel a part of the current social milieu and to attain or maintain high social status within the peer group were reported as prominent motives for substance use among affluent students in the current study. Given that the social milieu is crucial for the substance use behavior in this context, future research on substance use prevention targeting this group could with advantage pay attention to suggestions on prevention strategies given by the students. Students’ suggestions include reliable prevention information from credible sources, stricter substance control measures targeting those providing substances, parental involvement, leisure activities without substance use, and confidential ways to seek help, involving a non-judging approach from professionals and other adults.

## Supplementary Information

**Additional file 1.**

## Data Availability

Collected data will be available from the Centre for Psychiatry Research, a collaboration between Karolinska Institutet and Region Stockholm, but restrictions apply to their availability, as they were used under ethical permission for the current study, and so are not publicly available. However, data are available from the authors upon reasonable request and with permission from the Centre for Psychiatry Research.

## Abbreviations

ADHD: attention deficit hyperactivity disorder
Natural/tech/math: natural sciences/technology/mathematic programs
Social/hum: social sciences/humanities programs
STAD: Stockholm prevents alcohol and drug problems
