# Supplementary material for: How to prevent alcohol and illicit drug use among students in affluent areas: a qualitative study on motivation and attitudes towards prevention
Source: Subst Abuse Treat Prev Policy. 2021 Nov 7;16:83. doi: 10.1186/s13011-021-00420-8 (PMC8573923; doi:10.1186/s13011-021-00420-8)
Supplement: Supplementary file 1 — Additional file 1. [file 13011_2021_420_MOESM1_ESM.docx]

# Supplementary material

# **Interview guide**

**About the interview questions**

The interview questions are asked to the interviewee, and based on his or her answers, follow-up questions are asked. Questions that aim to specify the interviewee's answers, such as “can you clarify / develop what you just said” or “do you want to give more examples”, are also used, as well as questions to ensure the interviewer's correct understanding of interview answers (“do you mean that”? or “have I understood correctly?”). The latter contribute to the validation of the researcher's interpretations. In accordance with qualitative interview practice, the interview questions may be developed during the course of the study. The following is thus to be regarded as a starting point / illustration of the type of questions that may be asked.

**BACKGROUND INFORMATION**

*The purpose of these questions is to get an idea of ​​who the interviewee is and to start the interview with questions that are easy to answer to start the conversation.*

I would like to start by asking a few questions about you, about who you are. You are also welcome to tell me yourself if you want.

What gender, what age, what grade?

Which high school specialization have you chosen?

What do you think about school and school work? (Important to go to school / to pass school / to perform / get high grades?)

What does your family situation look like? (Parents, siblings, residents, etc.)

Relation to the place - Do you live in Municipality X? (some only go to school here)

How long have you lived in Municipality X? Previous accommodation?

What do your parents work with / what education do your parents have?

How often do you meet your parents? (Growing up in a two-career family or other family form)

What are your hobbies? (Important to perform in leisure time? What does leisure activity do for you?)

Have you, or have you previously, had contact with e.g. doctor, counselor, psychologist and nurse because of your mental health?

**ALCOHOL AND DRUG CONSUMPTION AMONG YOUNG PEOPLE**

*This theme is related to the question of what young people themselves consider to be the reason for the high alcohol consumption in Municipality X. Here they get to reason about "other young people" and their alcohol and drug habits.*

What does alcohol consumption look like among young people in Municipality X?

When do young people start using alcohol?

What do you think are the main reasons why young people drink?

Is there another reason why young people drink alcohol? (Ask them to reflect)

Are there any expectations that you as a young person should drink alcohol? If so, when does it start?

Is there reason to be concerned about young people's alcohol habits? Why?

How do you get hold of alcohol as a teenager? (Ask them to reflect on the most common / simplest / most accepted way)

What do you know about drug use among young people in Municipality X?

What type of drugs are used? Is it common? When do young people start taking drugs?

Why do young people use drugs?

**OWN RELATIONSHIP TO ALCOHOL AND DRUGS**

*This theme is about their own alcohol habits and what reasons they give for not drinking alcohol or drinking moderately or extensively.*

How would you describe your own relationship to alcohol? (Has it always been this way?)

Have you ever been drunk? (How often? Do you remember the first time, etc.)

For what reasons have you chosen to drink or drink moderately/abstain from alcohol?

Are there other reasons why you have chosen to give up (Ask them to reflect - school performance, leisure performance, lack of relationships, other things?)

What is the best thing about drinking moderately/abstaining from alcohol?

Are there times/situations where you would like to drink alcohol?

Is there anything negative about not drinking alcohol?

How would you describe your health? (For example, have you chosen to give up due to health reasons?)

Do you have experience of using drugs of any kind? (Ask them to elaborate on if they have tried drugs, at what age, what drugs, why, etc.)

**RELATION TO PEERS**

*The theme is related to what drinking habits look like among peers and how any distancing from alcohol is received by peers.*

How would you describe your social relationships with peers in and outside Municipality X?

Do you have a social network - ie. friends/friends - at school and/or in Municipality X?

If you think of your closest circle of friends - what do the alcohol habits/attitudes look like among them?

What characterizes your own group of friends - how would you describe yourself in relation to others? (How would outsiders describe your peer group, status, interests, values, etc.?)

Are you part of any peer groups where alcohol is common/unusual?

For those who abstain/drink moderately:

- How did your peers react to the fact that you chose to abstain/drink moderately?

- Have you ever been questioned by peers because you chose not to drink alcohol?
 (Ask them to tell you how)

- Have you ever received a positive response from peers because you have chosen to
 abstain from alcohol? (Ask them to tell you how)

- What reasons do you usually give to peers when you choose to abstain from
 alcohol? (How is it received, are there reasons that are rarely questioned - ask for
 tips)

-Statistics show that young people in Municipality X drink a lot of alcohol - What i

it like to be someone who refrains from drinking alcohol when many others drink?

(Peer pressure, etc.)

**ALCOHOL HABITS IN THE FAMILY**

*The theme is related to what the drinking habits looked like in the own family and what influence the family has for the individual's choice.*

What are the alcohol habits in your own family?

Do your parents drink alcohol - If so, how often?

Have you ever seen your parents drunk? (If yes - How often and how did you react?)

Have your parents talked to you about alcohol/drugs while you were growing up? (At what age?/In what way?)

How would your parents react if they found out you were drunk or taking drugs? (Has it happened? What happened/did they / did they do?)

How is your parents' attitude to you being allowed to taste/drink alcohol at home, e.g. to the party?

Have your parents ever bought/given alcohol to you (or siblings)?

Have your parents influenced your choice to drink/not drink alcohol? In what way?

Has anyone in your family or relatives had problems with alcohol or drugs?

**SUPPORT TO DECREASE ALCOHOL CONSUMPTION AMONG YOUNG PEOPLE**

*This theme is related to the question of how alcohol consumption can be reduced among young people in Municipality X.*

The goal is to get young people to drink less alcohol. (For those who abstain/drink moderately: In that respect you are a role model).

What do you think is the best way to get young people not to drink/drink less alcohol?

What would you do if you were worried about a friend drinking too much? (Eg. how to talk to her/him?)

What support and help do you think young people need from the adult world to reduce their alcohol consumption?

At what age are efforts against alcohol and drugs most needed? (Primary school/Secondary school or Elementary-/Middle-/High school)

Is the information about alcohol and drugs that parents, teachers, and researchers present relevant and credible? (Do you remember any information they received? Which source is most credible? Why/why not? What was good/bad? Other young people's reactions?)

What do you think needs to be done for more people to choose like you, ie. not to use alcohol and drugs?

**TERMINATION**

Is there anything that we have missed in this conversation about alcohol and drugs that you would like to add?

Is there anything else you want to bring up or ask about?

Do you know where to turn if you are worried about yourself or a friend?

After undergoing this interview, do you agree that this interview is saved and handled anonymously by the responsible researchers in the study?

Thank you for your participation.
